# An Estrogen–NK Cells Regulatory Axis in Endometriosis, Related Infertility, and Miscarriage

**DOI:** 10.3390/ijms25063362

**Published:** 2024-03-16

**Authors:** Shaoliang Yang, Haiyan Wang, Dajin Li, Mingqing Li

**Affiliations:** 1 Lab of Reproductive Immunology, Hospital of Obstetrics and Gynecology, Fudan University, Shanghai 200011, China; yangshaoliang7979@fckyy.org.cn (S.Y.); djli@shmu.edu.cn (D.L.); 2 Department of Gynecology of Integrated Traditional Chinese and Western Medicine, Hospital of Obstetrics and Gynecology, Fudan University, Shanghai 200011, China; wanghaiyan1431@fckyy.org.cn; 3 Shanghai Key Laboratory of Female Reproductive Endocrine Related Diseases, Hospital of Obstetrics and Gynecology, Fudan University, Shanghai 200011, China

**Keywords:** estrogen, NK cell, endometriosis, endometrial receptivity, infertility, embryo implantation, pregnancy loss

## Abstract

Endometriosis is a common estrogen-dependent condition that impacts 8–10% of women in their reproductive age, resulting in notable pain, morbidity, and infertility. Despite extensive research endeavors, the precise cause of endometriosis remains elusive, and the mechanisms contributing to its associated infertility are still not well comprehended. Natural killer (NK) cells, vital innate immune cells crucial for successful pregnancy, have been investigated for their potential involvement in the pathogenesis of endometriosis. Prior research has mainly concentrated on the diminished cytotoxicity of NK cells in endometrial fragments that evade the uterus. Interestingly, accumulating evidence suggests that NK cells play multifaceted roles in regulating the biology of endometrial stromal cells (ESCs), promoting local immune tolerance, influencing endometrial receptivity, oocyte development, and embryo implantation, thereby contributing to infertility and miscarriage in patients with endometriosis. In this comprehensive review, our goal is to summarize the current literature and provide an overview of the implications of NK cells in endometriosis, especially concerning infertility and pregnancy loss, under the influence of estrogen.

## 1. Introduction

Endometriosis is a common disease characterized by the presence of endometrial glands or stroma cells outside the uterus. While these lesions are typically localized in the pelvic region, they can also manifest in various other anatomical sites, including the bowel, diaphragm, and pleural cavity. Despite being a prevalent and non-malignant disorder, the presence of ectopic endometrial-like tissue and the ensuing inflammatory response can lead to a range of symptoms including dysmenorrhea, dyspareunia, chronic pain, as well as psychological comorbidities like depression and anxiety. In cases where the lesions affect the intestines or bladder, individuals may experience menstrual-related discomfort including diarrhea, painful bowel movements, and hematuria. Moreover, alterations in the pelvic microenvironment can contribute significantly to infertility in up to 50% of individuals with endometriosis. Endometriosis has an incidence rate of approximately 10% among women of reproductive age, significantly compromising the quality of life for those affected and representing a prominent cause of female infertility [1,2].

Endometriosis is known to be influenced by estrogen, a hormone crucial in its pathogenesis. Specifically, endometriotic tissues exhibit elevated local estrogen levels due to increased expression of p450 aromatase, an enzyme involved in estrogen production, and reduced activity of 17β-hydroxysteroid dehydrogenase type 2, an enzyme responsible for metabolizing estrogen into the less potent estrone [3]. This heightened estrogen milieu promotes the proliferation, migration, and invasion of ectopic endometrial cells while inhibiting their apoptosis [4]. Additionally, estrogen activates the cyclooxygenase-II (COX-2)–prostaglandin E2 (PGE-2) feedback loop, which is known to exacerbate inflammation and contribute to the pathology of endometriosis [5]. In terms of mechanism, the collaboration between estrogen and cytoplasmic inflammatory factors has been shown to inhibit apoptosis and promote the invasiveness of the lesions. Furthermore, evidence suggests that estrogen signaling may also impact immune cells, and dysregulation of the immune system has been implicated in the growth of endometriotic lesions, thereby contributing to the establishment, pathophysiology, and adverse reproductive outcomes associated with endometriosis [6,7].

Natural killer (NK) cells, which are essential components of the innate immune system, play pivotal roles in defending against viral infections and tumor development while supporting tissue homeostasis. Within the human endometrium, uterine NK cells represent the predominant leukocyte population, constituting approximately 30–40% of total leukocytes during the proliferative phase and up to 70% during the secretory phase [8]. A growing body of evidence suggests the involvement of NK cells in the pathogenesis of endometriosis and its associated infertility [9,10]. Significantly, women with endometriosis exhibit a noteworthy decrease in the cytotoxicity of NK cells found in peripheral blood, peritoneum, and peritoneal fluid, potentially influenced by factors present in the serum and peritoneal fluid [11,12]. However, the broader functions of uterine NK (uNK) cells within this context remain poorly understood. Moreover, considering the established role of uNK cells in normal pregnancy, it is plausible that the observed defects in uNK cells in endometriosis may account for the high incidence of infertility observed in affected patients.

## 2. The Proportion, Phenotype, and Function of NK Cells in Normal Endometrium and Endometriosis

### 2.1. Periodic Changes in NK Cells in Normal Endometrium

In normal menstruation, the endometrium undergoes periodic changes in response to cyclical fluctuations of ovarian-derived sex steroids, including proliferation, differentiation, and shedding. As a result, the proportion and phenotype of uterine natural killer (uNK) cells also vary during different phases. During the proliferative phase, only a small number of uNK cells are dispersed throughout the functional layer. However, after ovulation, there is a significant increase in uNK cell numbers, rapid proliferation, and acquisition of a granular phenotype in the luteal phase, reaching a peak a few days prior to menstruation [13,14,15]. By day 20 of the menstrual cycle, approximately 30% of uNK cells express Ki-67, indicating their strong proliferative response during endometrial regeneration. In contrast, the proportion expressing Ki-67 was less than 5% by day 7 [14]. In the late secretory phase, uNK cells constitute almost 70% of endometrial leukocytes [16]. As uNK cells surround the arteries and glands of the endometrium, they are shed with the menstrual blood during menstruation, leading to a higher proportion of NK cells in menstrual blood lymphocytes compared to peripheral blood [17]. Previous studies have reported that two days before menstruation, NK cell apoptosis occurs due to a decrease in progesterone levels, which leads to a decline in NK cell numbers at the start of a new cycle [18,19].

Human NK cells can be classified into two groups based on the expressions of CD56 and CD16: CD56^dim^CD16^+^ cells exhibit high cytotoxicity and function as killer cells to prevent pathogenic infection, whereas CD56^bright^CD16^−^ cells are characterized by potent cytokine secretion and multiple regulatory effects [20]. Previous observations have indicated that CD56^dim^ cells are prevalent in the proliferative phase, while CD56^bright^ cells are predominant in the secretory phase [21], indicating distinct functions of NK cells during these phases. During the late proliferative phase, endometrial NK cells demonstrate heightened cytotoxicity and upregulation of activation markers, including CD69 and HLA-DR, thus contributing to protective immunity against microbial infections [22]. Furthermore, during the secretory phase, uterine natural killer cells have been observed to express mRNA for vascular endothelial growth factor-C (VEGF-C), placental growth factor (PLGF), and angiopoietin-2 (Ang-2), indicating their potential role in preserving the stability of the uterine artery [22]. KIR^+^CD39^+^ uNK cells are thought to play a critical role in regulating fetal–maternal tolerance and vascularization through the production of galectin-1 and galectin-9. In the initial and intermediate phases of the menstrual cycle, there is a greater frequency of KIR^−^CD39^−^ uterine natural killer cells, while the populations of both KIR^+^CD39^−^ and KIR^+^CD39^+^ uNK cell subsets increase towards the end of the cycle. These findings suggest a potential involvement of NK cells in immune tolerance and enhanced angiogenic capacity during the late secretory phase [14]. (See in Table 1).

### 2.2. NK Cells in Endometrium, Peripheral Blood, and Peritoneal Fluid in Endometriosis

In women with endometriosis, eutopic natural killer (NK) cells exhibit an increase in the secretory phase similar to that of women without endometriosis, and the absolute number is reported to be comparable or lower [23,24]. However, eutopic NK cells in women with endometriosis exhibit lower cytotoxicity compared to those in women without endometriosis. Conversely, in women with endometriosis and infertility or recurrent pregnancy loss, an increase in the expression of NKP46 and CD16 has been reported [15,25]. Additionally, women with endometriosis have been found to exhibit an elevated proportion of immature uterine NK (uNK) cells at developmental stages 1 and 2 in the endometrium, indicating aberrant maturation of local uNK cell populations in endometriosis compared to normal controls [26]. Conversely, ectopic NK cells are consistently low throughout the menstrual cycle [24]. A recent single-cell study identified four distinct NK subpopulations in normal endometrium and ectopic lesions: three CD56^bright^ NKs (NK-1 with SELL and IL7R, NK-2 with ITGAX, and NK-3 without ITGAX) and one CD56^dim^ NK (NK-4 with highly cytotoxic effector gene expression of FGFBP2 and FCGR3A). Ovarian endometrial cysts and superficial peritoneal endometriosis predominantly exhibit the NK-3 subset of NK cells, which potentially contributes to immune regulation. Conversely, in deep infiltrating endometriosis (DIE), the NK-4 subset emerges as the dominant NK population, demonstrating the highest cytotoxicity scores [27].

In normal healthy women, peripheral NK cells change significantly throughout the menstrual cycle. The level of CD3^−^CD56^+^ NK cells is significantly higher in the mid-luteal (menstrual cycle day 20–22) and the late luteal (day 25–27) phases compared to the early follicular phase (day 2–4) [28]. In endometriosis, some studies have found that the number of peripheral NK cells does not significantly change or slightly decrease compared to normal women [9,28,29,30], while others reported that the percentage of peripheral NK cells increased, especially in patients with advanced stages of endometriosis (17.8% in stage III/IV) compared to those with early stages (11.8% in stage I/II) and normal controls (10.6%) [31]. Although there are different results regarding changes in the number of peripheral NK cells in endometriosis, it is unanimously agreed that peripheral NK cells have strong cytotoxicity; however, this effect is weakened in endometriosis. Research has shown that peripheral NK cytotoxicity is significantly decreased in endometriosis patients [32]. Oosterlynck et al. observed a decrease in the cytotoxic activity of peripheral NK cells in patients with stage I/II endometriosis as the disease progressed. However, in stage III/IV patients, although the cytotoxic activity of NK cells remained lower than that of the normal control, it was actually elevated compared to stage I/II patients [33]. Other studies have reported lower levels of CD56+NKG2D+ NK cells in ovarian endometrioma patients [34]. Furthermore, a marked decrease in TIM-3 expression on peripheral NK cells may also contribute to impaired immune surveillance in endometriosis [35].

There is a lack of direct research on changes in NK cells in the menstrual cycle in peritoneal fluid. Considering that the source and local microenvironment regulation of peritoneal NK cells are largely similar to uterine NK cells, we speculate that the changes in peritoneal NK cells are similar to uterine NK cells in normal women. Numerous studies have confirmed that the quantity of NK cells in the peritoneal fluid of patients with endometriosis was higher than that in normal women [33,36]. Oosterlynck et al. reported an increased number of NK cells in the peritoneal fluid of patients with endometriosis compared to normal controls, along with a decrease in their cytotoxic function. As the disease progresses, the decline in NK cell cytotoxicity becomes more pronounced, reaching its lowest level in stage III/IV patients. Meanwhile, the quantity of NK cells was highest in the early stages and decreased as the disease advanced, with NK cell numbers in stage III/IV patients being comparable to those of normal controls [33]. Another recent single-cell sequencing study reported that the cytotoxic activity of NK cells decreased while the chemotactic effect was elevated in the peritoneal fluid of endometriosis, providing support for the conclusions drawn by Oosterlynck et al. [36]. Other investigations have centered on the characterization of killer activating receptors (KARs) and killer inhibitory receptors (KIRs) in peritoneal NK cells. Notably, they have revealed diminished expression of NKP46, NKG2C, NKG2D, and various other activating receptors in peritoneal NK cells in endometriosis [37]. Concomitantly, there is an upregulation of inhibitory receptors such as CD94/NKG2A, KIR2DL1, leukocyte immunoglobulin-like receptor B1 (LILRB1), and LILRB2, which serve to dampen the cytotoxic activity of NK cells. This phenomenon contributes to the immune evasion exhibited by ectopic endometrial cells [9,38]. (See in Table 1).

## 3. Regulation of Estrogen on Differentiation of Endometrial NK Cells

The maturation of NK cells takes place in the bone marrow and thymus through a sequential process involving hematopoietic stem cells (HSCs), common lymphoid progenitors (CLPs), NK lineage-restricted progenitors (NKPs), and mature NK cells [39]. Uterine natural killer cells are generated from the differentiation of local NKPs or the migration of peripheral NK cells. However, the characteristics and functional properties of uNK cells are distinct from those of peripheral NK cells, as they are predominantly influenced by signals derived from the local tissue microenvironment.

There is evidence suggesting that estrogen plays a regulatory role in the recruitment, proliferation, differentiation, and function of uterine natural killer (uNK) cells through both direct and indirect effects [40]. Studies have shown the estrogen receptors, including estrogen receptor alpha (ERα) and wild-type ERβ1 and its variant form Erβcx/β2, were conservatively expressed in NK cells in mice and humans [41,42,43,44]. The impact of estrogen on NK cell recruitment, proliferation, differentiation, and function has been observed to vary across different studies. For instance, T Inoue et al. reported no significant impact on the proliferation of uNK cells following estrogen administration (endometrial leukocyte-rich fractions composed of leukocytes and ESCs, 17β-estradiol, 10^−8^ mol/L, cultured for 6 days) in the culture medium [45]. Conversely, tamoxifen, an anti-estrogen drug, clearly inhibited the estrogen-induced pro-proliferative activity of NK cells (YT-NI7 cell line and primary large granular lymphocytes, 17β-estradiol at 10^−8^, 10^−7^, and 10^−6^ mol/L, cultured for 48 h) [46]. In terms of uNK cell recruitment, Chantakru, S, demonstrated that estrogen could enhance homing (ovariectomized C57Bl/6 mice, 17β-estradiol at 100 ng/day, 3 days) by upregulating the expression of L-selectin and α-integrin on circulatory CD56^bright^ NK cells [47]. Conversely, Ma, R et al. found that estrogen stimulation (pseudopregnant CD1 mice obtained blastocysts transfer, intraperitoneal injection of 5 IU of Pregnant mare serum gonadotropin (PMSG) followed by 5 IU of hCG 48 h later) significantly reduced the density and maturity of uNK cells in pregnant mice, suggesting an inhibitory effect of upregulated estrogen signaling on the accumulation and maturation of uNK cells [48]. Regarding NK function, Jaillon et al. reported that pro-inflammatory cytokine production and NK cell activity increased after menopause, whereas estrogen therapy (0.625 mg conjugated estrogen + 2.5 mg medroxyprogesterone acetate for 2 months) could reverse this effect [49,50], indicating that estrogen may suppress peripheral NK cell activity and the secretion of pro-inflammatory cytokines. Furthermore, NK cells were found to increase after surgical menopause and estrogen at levels comparable to the ovulatory phase or pregnancy could suppress NK cell cytotoxicity and downregulate NK activating receptors (ovariectomized C57BL/6J mice received daily s.c. injections of 17β-estradiol, 100 μg/kg/day for 1 week) [51,52]. However, other studies revealed that estrogen treatment enhanced the cytotoxic activity of human NK cell lines against tumor cells (YT-NI7 cell line, 17β-estradiol at 10^−8^ and 10^−6^ mol/L, cultured for 48 h), an effect that could be blocked by tamoxifen, suggesting that estrogen potentiated NK cell killing function through ERα activation [46]. Estrogen was also reported to increase the secretion of CCL2 in uNK cells (primary uNK cells, estrone 10^−8^ mol/L or estradiol 10^−8^ mol/L, cultured for 2 h), which is involved in the construction of blood vessels in the endometrium [53]. These conflicting findings on NK cell proliferation, recruitment, cytotoxicity, and cytokine secretion [46,47,48,49,50,51,52] indicate that the influence of estrogen on NK cells may differ based on the cell type, the method of administration, and the duration of exposure to estrogen. This highlights the need for additional research to fully understand these complex interactions.

The influence of estrogen on uterine NK cells has been observed indirectly, mediated by neighboring cells. Experimental studies have revealed that estrogen can upregulate the expression of CXCL10 and CXCL11 in endometrial cells (endometrial tissue specimens, 17β-estradiol at 10^−9^, 10^−10^, and 10^−11^ mol/L, cultured for 48 h), which are involved in the regulation of NK cell recruitment during pregnancy [54]. In contrast, other studies have indicated that decidual stromal cells with estrogen stimulation (pseudopregnant CD1 mice obtained blastocysts transfer, intraperitoneal injection of 5 IU of PMSG followed by 5 IU of hCG 48 h later) exhibited reduced expression of various chemokines and cytokines, such as CXCL12, CXCL14, CX3CL1, IL-15, PLGF, and TGF-β. These findings suggest the presence of abnormal estrogen signaling during ovarian stimulation prior to assisted reproductive technologies [48]. HAND2, a transcription factor implicated in the cellular response to estradiol, exerts direct regulatory control over the expression of IL-15 in human ESCs. This molecular interaction plays a pivotal role in facilitating the development and maturation of NK cells [39,55]. Additionally, since ERα expression is shared by several immune cells, they can also act as intermediaries in influencing NK cells. These findings suggest that estrogen signaling may impact the surrounding cells in the endometrium, thereby regulating the recruitment, proliferation, and differentiation of uNK cells.

## 4. Estrogen–NK Cell Axis in Endometriosis

### 4.1. The Cytotoxic Effect of NK Cells on ESCs Mediated by Estrogen

Endometriosis is an estrogen-dependent inflammatory disease characterized by excessive estrogen signaling within endometriotic lesions. This is attributed to elevated estrogen production, inadequate estrogen metabolization, and altered expression of estrogen receptors. Upregulation of ERβ and downregulation of ERα in ectopic endometrium suggest that ERβ functions as the primary receptor for estrogen in the endometrium. Activation of ERβ by estrogen stimulates the expression of PGE2 and Ras-like, estrogen-regulated, growth inhibitor (RERG), thus promoting the proliferation of enESCs. Furthermore, other signaling pathways, including c-MYC, Greb-1, and FGF-9, have been linked to estrogen-mediated ESC growth [3,56].

Although the impact of estrogen on the cytotoxic activity of NK cells remains controversial across various models, most studies suggest compromised cytotoxicity of local NK cells towards ESCs in endometriosis [57]. Studies have shown that estrogen can suppress the cytotoxicity of NK cells, as demonstrated in our previous research, which revealed reduced expression of Granzyme B and NKG2D in peritoneal NK cells in mice [58]. Moreover, our findings indicate that protopanaxadiol, a ginseng extract structurally similar to estrogen, acts as an estrogen antagonist and significantly enhances NK cell cytotoxicity through the estrogen receptor (ESR) in mice. This enhancement may be attributed to the induction of autophagy in ESCs, a process suppressed by estrogen [58,59]. Furthermore, our results demonstrate that estrogen-mediated suppression of autophagy in ESCs impedes the differentiation of CD16^+^ NK cells, affecting the expression of COX-2 and the secretion of cytokines IL-8 and IL-23A in endometriosis. As a result, this inhibition compromises the cytotoxicity of NK cells [60]. In conclusion, estrogen can hinder the cytotoxicity of NK cells and promote immune evasion by ESCs, possibly through its involvement in suppressing autophagy.

### 4.2. The Regulatory Effect of Estrogen–NK Cells on ESC Adhesion, Migration, and Invasion

Ectopic ESCs exhibit invasive, recurrent, and metastatic properties, making them a valuable model for comparison with trophoblast and tumor cells. Although the crucial role of decidual NK cells in regulating the invasive function of trophoblast cells during early pregnancy is well-established, there is limited literature on the regulatory role of NK cells in ESC invasion in endometriosis. Specifically, there are few reports that directly investigate the effect of NK cells on ESC adhesion, migration, or invasion. However, it is worth noting that several studies have shown the simultaneous regulation of NK function and ESC biological behaviors within the local microenvironment. In the context of endometriosis, elevated estrogen levels promote IL-15 production in ectopic endometrium, thereby stimulating the growth and invasion of ESCs and suppressing the cytotoxic activity of NK cells [61,62]. Furthermore, estrogen-induced Galectin-3, an immunoregulatory protein, has been shown to enhance peritoneal engraftment of ESCs by impairing immune surveillance of NK cells in the peritoneal cavity, ultimately promoting ESC adhesion and migration to this site [63,64]. Additionally, IL-17A, a pro-inflammatory cytokine closely associated with the estrogen–COX-2 axis, has been found to promote the survival of endometrial cells through upregulation of anti-apoptotic genes. Treatment with IL-17A has also been reported to inhibit NK cell-mediated cytotoxicity and induce HLA-G expression in endometrial cells [65]. Taken together, these studies suggest that NK cell function is intricately linked to the biological behaviors of ESCs, akin to the regulatory role NK cells play in trophoblasts during early pregnancy. However, due to the scarcity of direct evidence, further investigation is warranted to comprehensively understand the intricate interaction between NK cells and ESCs in regulating these biological behaviors.

### 4.3. The Regulatory Effect of Estrogen–NK Cells on Vascularization of Ectopic Lesion

Angiogenesis, a critical process in the growth and development of ectopic lesions in endometriosis, involves a complex and multistage progression. This includes the activation of endothelial cells, degradation of the basement membrane, directional migration and sprouting, tube formation, maturation, and remodeling. Various cell types participate in regulating angiogenesis by secreting stimulators or inhibitors to control this process. Women with endometriosis exhibit disrupted angiogenic activity in their eutopic endometria. This is characterized by increased endothelial cell proliferation, higher microvessel density, and elevated levels of VEGF-α, Ang-1, and Ang-2 compared to healthy women [66,67].

Estrogen has been recognized to play a significant role in the angiogenic activity of patients with endometriosis [68]. However, our understanding of the impact of NK cells on the production of proangiogenic cytokines remains limited. Nonetheless, it has been observed that NK cells present at the decidua can influence placental angiogenesis through the production of VEGF, PLGF, interleukin (IL)-8/CXCL8, IL-10, Ang-1, and Ang-2, thereby contributing to vascularization during embryonic and placental development [67,69]. Additionally, research has indicated that NK cells in the normal secretory phase of the endometrium exhibit intense hybridization for VEGF-C, PLGF, and Ang-2 mRNAs, suggesting their involvement in promoting angiogenesis outside of pregnancy. Estrogen has also been found to stimulate uterine NK cells to secrete CCL2, further enhancing angiogenesis in ESCs [53]. As the local concentration of estrogen increases within endometriosis lesions, the angiogenic potential of ectopic NK cells is likely to be further enhanced. Moreover, IL-15, which shows high expression in ectopic endometrium and is regulated by estrogen, can significantly upregulate VEGF-C expression in NK cells [22,70], indicating an abundance of VEGF-C in NK cells within endometriosis lesions. Therefore, it is plausible to propose that NK cells in the local microenvironment of endometrial lesions play a role in promoting angiogenesis.

### 4.4. The Role of the Estrogen–NK Cell Axis in Immune Regulation in Endometriosis

**Figure 1:**
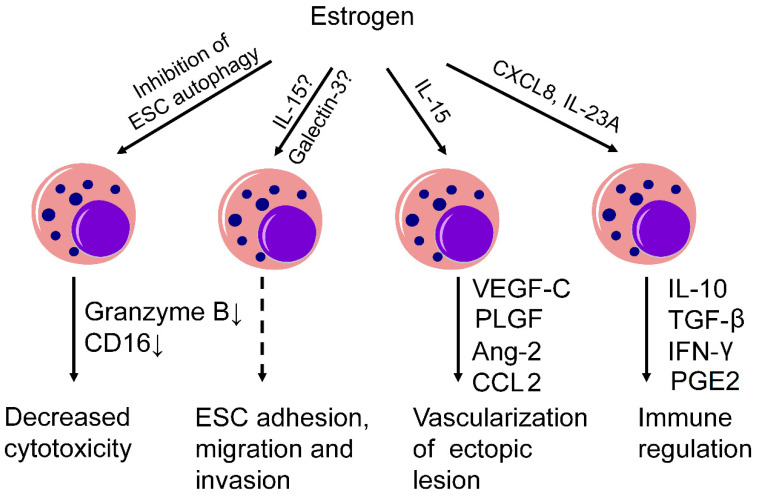
The estrogen–NK cell axis in endometriosis: Estrogen-mediated NK cells play a pivotal role in the clearance of endometriotic lesions, regulation of ESC biology, angiogenesis, and modulation of the local immune microenvironment. The estrogen-mediated suppression of autophagy in ESCs could impair NK cell cytotoxicity by downregulating the expression of CD16 and Granzyme B. The function of NK cells is closely associated with ESC adhesion, migration, and invasion, which may be regulated by IL-15 or Galectin-3. Estrogen may enhance the vascularization of ectopic lesions through cytokines released by NK cells. Additionally, estrogen-regulated NK cells can induce immunotolerance through IL-10, TGF-β, IFN-γ, and PGE2. (Solid arrows: supported by research findings; dashed arrows: inferred from current research, which lacks direct evidence; ESC: endometrial stromal cells; VEGF-C: vascular endothelial growth factor-C; PLGF: placental growth factor; Ang-2: angiopoietin-2; PGE2: prostaglandin E2). ↓: decreased, ?: need further evidence.

The dysfunction of the peritoneal immune microenvironment is a significant contributing factor to the persistence, implantation, and growth of ectopic endometrial tissue within the peritoneal cavity. As essential components of the innate immune system, NK cells regulated by estrogen closely interact with other immune cells to collectively establish the immune microenvironment at the site of the local lesion. Our previous study reported that estrogen could induce COX-2^+^CD16^−^ NK cell differentiation in endometriosis, which highly expressed IL-10 and TGF-β [27]. As a result, the high level of PGE2 produced by COX-2^+^ NK cells may induce the differentiation of Treg cells, promote their function, and further increase the survival, migration, and invasion of endometriotic cells [71]. Additionally, the anti-inflammatory cytokines IL-10 and TGF-β can suppress the proliferation of CD4^+^CD25^−^ T cells [72], inducing immunotolerance and contributing to the evasion of ectopic endometrial cells. We have also found that PPD, as a competitive antagonist of estrogen, can downregulate the expression of IL-10 and upregulate the expression of IFN-γ in NK cells, indicating the regulatory effect of estrogen on IL-10 and IFN-γ expression in NK cells [58]. Consistent with this, Fukui et al. also reported an increase in the production of IFN-γ in peritoneal fluid NK cells in endometriosis [73], which could further regulate the balance between Th1/Th2 and the differentiation of Treg/Th17 cells in peritoneal fluid [74]. (Shown in Figure 1).

Collectively, the estrogen–NK axis contributes to the persistence of inflammation, impaired immune surveillance, and the establishment of endometriotic lesions. However, further research is warranted to fully elucidate the mechanisms underlying their implications in endometriosis.

## 5. Estrogen–NK Cells in Endometriosis-Related Infertility and Miscarriage

### 5.1. The Role of Estrogen–NK Cells in Abnormal Ovarian Follicular Development

During the growth and maturation of antral follicles, the oocyte undergoes a series of processes to reach the optimal stage for fertilization. Concurrently, the formation of an antrum within the follicle leads to the accumulation of follicular fluids containing various components. As one of the most prominent components of follicular fluid, estrogen is dominant between the mid-follicular and pre-ovulatory phases [75]. Through different estrogen receptors, it plays important roles in follicular development by inhibiting ovulation, stimulating follicular growth, decreasing atresia, and inducing specific gene expression [76]. While the concentration of estrogen in follicular fluid was reported to be the same in endometriosis patients and control patients [77], the downstream signals of estrogen remain unconfirmed by current research.

As reported, the proportion of NK cells and their cytotoxicity in endometriosis were significantly increased in follicular fluids [78], which was closely associated with adverse reproductive events due to their negative impact on folliculogenesis and oocyte maturation. Except for the direct cytotoxicity towards oocytes, NK cells may influence oocyte development by elevated concentrations of IL-6, IL-8, and IL-12 [79]. IL-8 was involved in the recruitment, activation, and proliferation of neutrophils, and IL12 was reported to induce the Th1 response and regulate the biological activities of T cells, which were also associated with adverse pregnancy outcomes in follicular fluids [79]. The abnormal activation of NK cells and the proinflammatory milieu they induce within follicular fluids have been linked to adverse reproductive outcomes in endometriosis. This association stems from their detrimental impact on the processes of folliculogenesis and oocyte maturation.

Although studies have shown a close association between NK cells in follicular fluid and aberrant follicular development and infertility in endometriosis, direct evidence confirming the regulatory role of estrogen in this process is currently lacking. Therefore, we have made reasoned speculations based on some relevant research findings. Our previous research has confirmed that estrogen can downregulate the expression of CD16 on NK cells in endometriosis [60], with CD16 being one of the most prominently altered markers on NK cells in the follicular fluid of endometriosis. Considering that estrogen concentration in follicular fluid can escalate to over 1000 times the concentration in serum, significantly higher than the concentrations utilized in our experiments, the effect could be different as CD16 increases in the follicular fluid of endometriosis. While no significant changes in estrogen levels have been observed in follicular fluid between normal women and endometriosis patients, the alteration of downstream signals of estrogen remains unconfirmed. Further investigation is required to elucidate the role of estrogen in the interaction between NK cells and follicular development.

### 5.2. The Estrogen-Regulated Embryotoxicity of NK Cells in Peritoneal Fluid

The embryotoxicity of peritoneal fluid is a significant contributor to infertility in individuals with endometriosis. Multiple studies have demonstrated that the peritoneal fluid of affected patients markedly inhibits the fertilization capability of oocytes and the development potential of embryos [80,81,82]. The presence of cytokines such as TNF-α, IFN-γ, and IL-6 in the peritoneal fluid can induce cellular stress, compromise cell viability, and impede blastocyst development. Moreover, elevated concentrations of these cytokines can lead to embryo demise. Experiments in mice have demonstrated that these cytokines exert detrimental effects on embryos through pro-apoptotic and adverse programming effects. Additionally, they indirectly suppress uterine receptivity by eliciting a maternal immune response [83,84].

NK cells have emerged as a pivotal subset of cells responsible for the embryotoxicity of peritoneal fluid in patients with endometriosis. Elevated levels of NK cells and IL-6 have been observed in the peritoneal fluid of endometriosis patients, which correlates with increased embryotoxicity [81]. Furthermore, NK cells secrete various cytokines and have been identified as the primary source of TNF-α in peritoneal fluid [85]. Notably, TNF-α played a critical role in mediating the embryotoxicity of peritoneal fluid in endometriosis patients, mimicking its effects, and can be effectively mitigated by TNF-α inhibitors [86]. These findings provide strong evidence for the crucial involvement of NK cells in the embryotoxicity of peritoneal fluid in patients with endometriosis.

Despite the absence of direct evidence, previous studies suggest that estrogen may regulate the NK cell-mediated embryotoxicity of peritoneal fluid in endometriosis patients by modulating pro-inflammatory cytokine secretion by NK cells [87]. Our prior experiments demonstrated that estrogen and PPD can affect cytokine expression, particularly IFN-γ, in NK cells [58], which suggests a potential involvement of estrogen in regulating NK cell embryotoxicity. Further investigations are warranted to uncover the specific role and mechanisms underlying this effect.

### 5.3. Implantation Failure Caused by Inadequate Function of Estrogen–NK Cells in Endometriosis

Endometrial receptivity is a critical factor determining the success of pregnancy in patients with endometriosis. In a normal endometrium, prior to embryo implantation, NK cells secrete pro-inflammatory cytokines such as IL-6, IL-8, and PROK1, inducing a local inflammatory environment that facilitates successful implantation of the embryo. Subsequently, post-implantation, the local endometrium rapidly transitions from a pro-inflammatory environment to an anti-inflammatory one, with a buildup of dNK cells secreting IL-25, inducing decidualization of ESCs and vascular remodeling, thus promoting a smooth progression of pregnancy [88]. However, in endometriosis, estrogen-mediated signaling abnormalities may lead to decreased endometrial receptivity, impacting embryo implantation and reducing pregnancy rates in mice [89,90]. It was also reported that immunological and inflammatory mechanisms in endometriosis have a significant additive negative impact on embryo implantation [91]. Within this process, NK cells may play a crucial regulatory role.

A recent single-cell transcriptome study has shed light on the characteristics of endometrial cells during the proliferative phase and window of implantation (WOI) in both normal controls and patients with endometriosis. The results indicate that in normal endometrium, the percentage of NK cells during WOI significantly increases from 30% to 50%, whereas in endometriosis, NK cells conversely decrease [92]. Moreover, the expression of RANTES and other receptor–ligand pairs differs between normal endometrium and endometriosis, suggesting altered cross-talk between NK cells and other cells such as immune cells, epithelium, and stromal cells, which may influence the local environment and endometrial receptivity. As estrogen played indispensable roles in regulating the migration and recruitment of NK cells and could promote the secretion of CCL2 and other cytokines [53,54], we speculated the abnormal number and phenotype of NK cells were related to estrogen. In our previous study, we presented evidence supporting the positive effects of PPD, as a competitive antagonist of estrogen, expressed a notable improvement in the pregnancy rate and embryo implantation numbers, accompanied by a reduction in embryo abortion. We revealed that PPD exerted an up-regulatory influence on the expression of Ki67, VEGF, TGF-β, and CXCL10 in mice decidual NK cells. These results strongly indicate a potential association between endometrial receptivity, embryo implantation, and the residence and differentiation of decidual NK cells regulated by estrogen signaling [59].

### 5.4. The Relationship between NK Cells and Pregnancy Loss in Endometriosis

The association between endometriosis and the risk of miscarriage has been a topic of debate for many years, and various studies have yielded different results. A recent nationwide cohort study involving 29,563 women with endometriosis and 295,630 controls demonstrated a significant association between endometriosis and pregnancy loss, as well as recurrent pregnancy loss. The strength of this association increased with the number of losses, with adjusted odds ratios of 1.37 (95% CI: 1.32–1.42), 1.75 (1.62–1.89), and 2.57 (2.31–2.85) for 1, 2, and ≥3 pregnancy losses, respectively [93]. Similarly, Vercellini et al. found that patients with endometriosis had lower pregnancy and live birth rates, as well as an increased risk of miscarriage. This effect was more pronounced in women with adenomyosis than in those with endometriosis [94]. However, Manieri et al. challenged these findings, suggesting that the high proportion of IVF procedures among women with endometriosis, and the lack of association between the severity of endometriosis and the risk of pregnancy loss in the study could have biased the results [95]. In fact, a study by Simón et al. showed that endometriosis patients who underwent IVF using donor oocytes had similar rates of implantation and pregnancy compared to control recipients. However, when analyzing outcomes based on the source of donated oocytes, patients who received embryos derived from endometriotic ovaries had a significantly lower implantation rate, suggesting that oocytes rather than recipients were the key determinant of successful implantation [96]. Yu Wang also reported that endometriosis was not associated with adverse pregnancy outcomes in patients undergoing IVF, while adenomyosis was associated with higher miscarriage rates and lower live birth rates [97].

**Figure 2:**
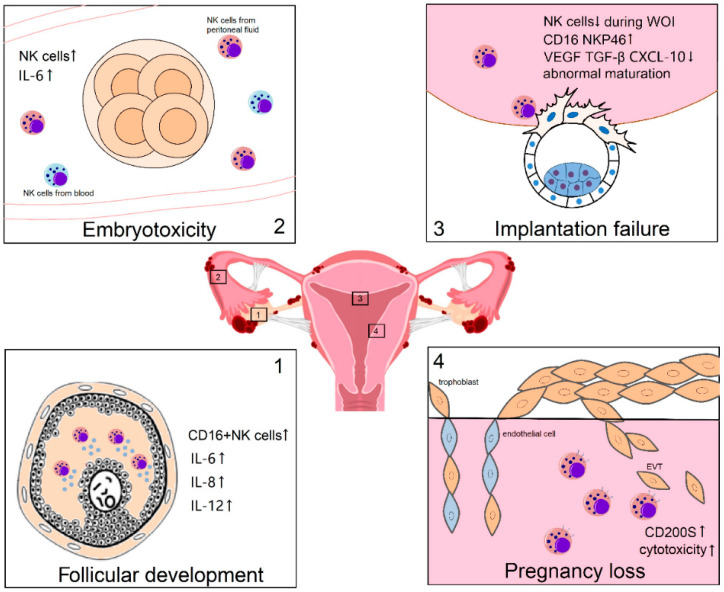
The role of NK cells in endometriosis-related infertility. NK cells play pivotal roles at various stages of infertility associated with endometriosis. Throughout follicular development, NK cells have the capacity to impede oocyte maturation through direct action and the secretion of cytokines. During the process of embryo transportation, NK cells derived from peritoneal fluid and blood may collaboratively exert embryotoxic effects. The implantation process witnesses a disturbed dynamic alteration in the quantity of NK cells, resulting in compromised NK cell maturation and heightened cytotoxicity, consequently leading to decreased uterine receptivity and subsequent implantation failure. Moreover, during early pregnancy, the elevated expression of CD200S on NK cells led to enhanced cytotoxicity, potentially mediating the immune rejection of the embryo. These factors collectively contribute to the infertility and miscarriage observed in patients with endometriosis. (WOI: window of implantation; EVT: extravillous trophoblasts). ↑: increased, ↓: decreased.

Although there is an ongoing debate regarding whether endometriosis patients have an increased risk of miscarriage, we have nevertheless provided a brief summary of the role of the estrogen–NK cell axis in miscarriage among patients with endometriosis. After successful embryo implantation and completion of decidualization, decidual NK cells continue to maintain pregnancy through the induction of local immune tolerance, promotion of uterine vascular remodeling, regulation of trophoblast invasion, and facilitation of embryonic development [88]. However, impaired functionality in these aspects may lead to miscarriage. CD200S, which has been associated with fetal rejection and miscarriage in early pregnancy, has been found to be expressed by NK cells in the endometrium. The frequency of CD200S-expressing NK cells is increased in endometriosis, potentially impacting embryo survival and leading to miscarriage [98]. Lai et al. also reported enhanced cytotoxicity and reduced angiogenesis-promoting effects of NK cells regulated by estrogen antagonists in endometriosis, which may contribute to pregnancy loss in vivo [59]. Giuliani et al. observed a higher number of cytotoxic CD16^+^ NK cells and NKp46^+^CD56^+^ cells in patients with infertility or recurrent pregnancy loss in patients with endometriosis [25]. (Shown in Figure 2).

## 6. Expectations

During the menstrual cycle, retrograde menstruation occurs in approximately 80% of women, but only around 10% of women develop endometriosis. This discrepancy is attributed to the robust immune surveillance function of the immune system, which identifies and eliminates ectopic tissues and cells to maintain the stability of the local microenvironment. In this process, NK cells play a pivotal role. In healthy women, peritoneal NK cells express CD56^dim^CD16^+^ and exhibit heightened expression of activatory receptors involved in killing. However, in patients with endometriosis, influenced by increased concentrations of local estrogen, the quantity and phenotype of NK cells in the peritoneal fluid, ectopic lesions, and even peripheral blood undergo alterations. Consequently, the impaired function of NK cells hinders the effective clearance of ectopic cells, thereby promoting lesion development.

Existing research indicates that NK cells in the peritoneal fluid of individuals with endometriosis demonstrate a tolerant phenotype and significantly reduced cytotoxic activity. This phenotype closely resembles that of decidual NK cells during early pregnancy. Decidual NK cells are the most prevalent immune cells during pregnancy, and their functionality has been extensively studied. However, investigations regarding the regulatory role of NK cells in endometriosis remain limited, as studies have primarily focused on their cytotoxic effects. Tolerant NK cells should possess potent cytokine secretion capabilities; however, relevant research relating to the regulatory effect of NK cells on surrounding cells is lacking, and the underlying molecular basis has yet to be revealed. Therefore, we believe that a deeper understanding of the role of NK cells holds significant implications for elucidating the pathogenesis of endometriosis.

Furthermore, NK cells exhibit diverse functions in endometriosis-associated infertility, including the regulation of follicle development, fertilized eggs, and endometrial receptivity, which contribute to implantation failure and miscarriage. Considering the cyclical variations in the quantity and functional phenotype of uterine NK cells regulated by estrogen, it is crucial to assess uterine receptivity by simultaneously evaluating the expression of genes associated with endometrial receptivity and the quantity and functional phenotype of NK cells before embryo implantation. This approach can provide a theoretical foundation for strategically timing embryo transfer, particularly for patients with endometriosis. Moreover, given the crucial role of the estrogen–NK axis in endometriosis and related infertility, modulation of key targets within this axis represents a potential therapeutic approach for addressing endometriosis and related infertility.

## Figures and Tables

**Table 1:** The proportion, phenotype, and function of NK cells in endometriosis.

	Proliferative	Secretory	Menstrual	Main Phenotype	Function
Normal endometrium	+	+++	++	CD56^high^CD16^low^	Cytokine is productive, promoting pregnancy [13,14,15,23]
Eutopic endometrium	+	+++	++	CD56^bright^CD16^−^, more immature	less cytotoxicity in general, more cytotoxic in EMs patients with infertility [15,23,24,25,26,27]
Ectopic lession	+	+	+	CD56^bright^ in ovarian cyst, CD56^dim^ in DIE	Immunoregulatory in endometriosis, highly cytotoxic in DIE [24,27]
Normal peripheral blood	+	++	+	CD56^low^CD16^high^	highly cytotoxic [28]
EMs peripheral blood	Similar or slightly changed to normal			CD56^dim^CD16^+^, increased KIR	diminished cytotoxic [9,28,29,30,31,32,33,34,35]
Normal peritoneal fluid	+	+++	++	CD56^dim^CD16^+^, Granulysin^high^	Cytotoxic [36]
EMs peritoneal fluid	higher than normal			CD56^bright^CD16^−^, KIR2DL1↑NKG2D↓	Decreased cytotoxicity, elevated pro-inflammatory and chemotactic effects [33,36,37,38]

+/++/+++: relative number of NK cells; ↑: increased, ↓: decreased; DIE: Deep infiltrating endometriosis.

## Data Availability

This study did not generate any new data. All the data presented in this review were obtained from original articles referenced in the text.
